# The Significance of Human Papillomavirus Receptors Related Genetic Variants in Cervical Cancer Screening

**DOI:** 10.1128/spectrum.05117-22

**Published:** 2023-06-26

**Authors:** Hongyu Xie, Mingjing Wei, Lifang Yao, Yi Liu, Xing Xie, Xiao Li

**Affiliations:** a Zhejiang Provincial Key Laboratory of Precision Diagnosis and Therapy for Major Gynecological Diseases, Women’s Hospital, School of Medicine Zhejiang University, Hangzhou, China; b Clinical Research Center, Women’s Hospital, School of Medicine Zhejiang University, Hangzhou, China; c Cancer Research Institute of Zhejiang University, Hangzhou, China; d Department of Gynecologic Oncology, Women’s Hospital, School of Medicine Zhejiang University, Hangzhou, Zhejiang, China; e Department of Obstetrics and Gynecology, Shaoxing Maternal and Child Health Hospital, Shaoxing, Zhejiang, China; f Hangzhou Xixi Hospital, 2 Hengbu Street, Liuxia Town, Xihu District, Hangzhou City, Zhejiang Province, China; Oklahoma State University College of Veterinary Medicine

**Keywords:** cervical cancer, HPV receptor, single nucleotide polymorphisms, HPV susceptibility, cervical intraepithelial neoplasia

## Abstract

To investigate the relationship between single nucleotide polymorphisms (SNPs) in human papillomavirus (HPV) receptor associated genes and HPV susceptibility and clinical outcomes in Chinese women, from October 2016 to March 2020, a total of 3,066 women were recruited for a 3-year prospective population-based cervical cancer screening clinical trial. The primary endpoint was histological cervical intraepithelial neoplasia 2 and worse (CIN2+). Twenty-nine SNPs of HPV receptor associated genes on women with available cytology residual samples at baseline were detected using MALDI-TOF MS. Eligible data were available for 2,938 women. Rs16894821 (GG versus AA, OR =1.71 [1.08 to 2.69]) and rs724236 (TT versus AA, OR = 1.73 [1.14 to 2.62]) in *SDC2* were significantly related to the HPV susceptibility. And rs2575712 (TT versus GG, OR = 2.78 [1.22 to 6.36]) in *SDC2* was associated with increased HPV 16/18 susceptibility. Four SNPs (rs1047057 and rs10510097 in *FGFR2* gene, rs2575735 in *SDC2* gene, and rs878949 in *HSPG2* gene) were significantly associated with persistent HPV infection. Importantly, the genotypes of rs16894821 under recessive model (GG versus AA/AG, OR = 2.40 [1.12 to 5.15]) in *SDC2* and rs11199993 under dominant model (GC/CC versus GG, OR = 1.64 [1.01 to 2.68]) in *FGFR2* were significantly associated with the disease progression. Finally, SNPs showed comparable efficacy in detecting CIN2+ for the women infected with non-HPV16/18 compared with cervical cytology (sensitivity: 0.51 [0.36 to 0.66] versus 0.44 [0.30 to 0.60], specificity: 0.96 [0.96 to 0.97] versus 0.98 [0.97 to 0.99], positive predictive value: 0.23 [0.15 to 0.33] versus 0.33 [0.22 to 0.47], and negative predictive value: 0.99 [0.98 to 0.99] versus 0.99 [0.98 to 0.99]). SNPs in HPV receptor related genes may influence HPV susceptibilities and clinical outcomes in Chinese women.

**IMPORTANCE** Virus receptors are known to mediate virus attachment and further lead to virus infection of the host cells. In the current study, we investigated the relationship between single nucleotide polymorphisms (SNPs) in human papillomavirus (HPV) receptor associated genes and HPV susceptibility and clinical outcomes in Chinese women, and to explore the new triaging strategy for non-16/18 high-risk HPV infection.

## INTRODUCTION

Cervical cancer (CC) is the fourth leading cause of worldwide cancer death for women with more than 300,000 deaths annually (1). High-risk human papillomavirus (HPV) infection, especially persistent HPV infection, has been recognized as the principal cause of CC (2). It is a continuous process from HPV infection to cervical intraepithelial neoplasia (CIN) and finally to invasive disease. To date, HPV vaccination and CC screening are considered the best methods to decrease the incidence and mortality of CC (3). Although vaccination is effective in preventing CC, poor awareness of vaccination, anxiety about adverse reactions to the vaccine, and practical issues such as short supply limit its wide application in China (4). So CC screening remains an effective way for early detection of precancerous lesions and cancers. HPV-based primary screening strategy was recommended for CC screening by the US Preventive Services Task Force in 2018 (5). However, because only a small proportion of patients with HPV infection will go on to develop CC, further triaging strategies are necessary so as to avoid overdiagnosis and overtreatment with primary HPV testing alone. Therefore, further identifying more effective biomarkers associated with persistent HPV infection and disease progression would contribute to a precise CC screening strategy for the secondary prevention of CC.

As we know, the development of CC is a complex, multistep process, including HPV infection, genetic, and environmental factors (6, 7). Virus receptors are known to mediate virus attachment and further lead to virus infection of the host cells, for example, signaling lymphocyte activation molecule (SLAM) and CD6 for measles virus (8), and vitamin D receptor (VDR) for Hepatitis C virus (9). HPV infection related receptors include EGFR, HSPG2, FGFR2, TSPAN1, and SDC2 (10–12), and HPV enters the host cell by binding the viral particles to the cell surface receptor. Genomic sequencing studies have revealed that CC was associated with various genome structural variations, deletion, insert-deletion, and copy number variations (13–15). Genetic polymorphism has been widely validated as one important impact factor for tumorigenesis and clinical outcomes of cancer (16–18). Single nucleotide polymorphism (SNP) is a variation in sequence with a frequency of greater than 1% in at least one population, and tag SNP representative SNP in a region of the genome with high linkage disequilibrium (19, 20). Chen et al. performed a genome-wide association analysis of single nucleotide polymorphisms (SNPs) and found that 3 novel loci in the major histocompatibility complex might affect susceptibility to CC and increase the risk of tumor development (17). Łaźniak et al. revealed the relationship between rs6983267 in CCAT2 and the progression of CC in the Polish population (21). Our previous cross-sectional study showed that a series of SNPs in the HPV receptor related genes were significantly associated with HPV susceptibility and disease progression (12). For further validating the associations of SNPs and HPV susceptibility, persistent HPV infection, and disease progression, 29 SNPs in HPV receptor related genes were chosen for evaluation in a prospective cohort study, hoping to explore the potential of SNPs as an objective indicator for triage strategy in CC primary screening.

## RESULTS

### Basic characteristics.

Totally 2,938 women with sufficient cytology samples received SNP detection, and 23.11% (679/2,938) women were lost after 3 years of follow-up. Among 313 women with HPV infection at baseline, 57 were HPV 16/18 positive and 256 were non-16/18 HPV positive. Baseline characteristics of 2938 participants were listed in Table S2. Among 255 women with HPV positive at 3rd year, 160 women were newly infected who were HPV negative at baseline, while 95 were persistent HPV infection after excluding loss of follow-up. Among HPV positive at baseline, 107 women were spontaneously cleared at 3rd year and 62 cases were diagnosed as CIN2+. Totally 66 patients were diagnosed as CIN2+ during 3 years (30, 3, 1, 32 CIN2+ at baseline, 1st, 2nd, and 3rd visit separately) and all of them were HPV positive at diagnosis.

### Associations between SNPs and HPV susceptibility.

The genotype distribution of 29 SNPs was in Hardy-Weinberg equilibrium (*HWE*) (*P* > 0.05). The SNP’s genotype and allele distribution were analyzed between groups with and without HPV infection (Table 1 and S3). GG genotype of rs16894821 in *SDC2* had significantly increased HPV susceptibility compared with AA genotype (odds ratio (*OR*) = 1.71, 95% confidence interval (*CI*) = 1.08-2.69, *P* = 0.0473). Similarly, the carriers of allele G had significantly increased HPV susceptibility compared with allele A (*OR* = 1.26, *CI* = 1.04-1.52, *P* = 0.0181). Further analysis demonstrated that the recessive model for rs16894821 (GG versus AA/AG, *OR *= 1.60, 95% *CI *= 1.03-2.50, *P* = 0.0377) was a significant association with HPV infection instead of the dominant model. TT genotype of rs724236 in *SDC2* had significantly increased HPV susceptibility compared with AA genotype (*OR *= 1.73, 95% *CI *= 1.14-2.62, *P* = 0.0285). Similarly, the carriers of allele T had significantly increased HPV susceptibility compared with allele A (*OR *= 1.28, 95% *CI *= 1.07-1.54, *P* = 0.0077). We also found a significant increase in HPV susceptibility both under the dominant model (AT/TT versus AA, *OR *= 1.29, 95% *CI *= 1.02-1.63, *P* = 0.0333) and recessive model for rs724236 (AA/AT versus TT, *OR *= 1.60, 95% *CI *= 1.07-2.391, *P* = 0.0226).

**TABLE 1:** Genotype and allele frequencies that were significant differences between HPV (+) and HPV (-) groups

SNP	HPV (+) (*n* = 313)	HPV (-) (*n* = 2625)	*OR* (95% *CI*)	*P* value
rs16894821				
Genotype				
AA	173 (55.27%)	1596 (60.80%)	Reference	
AG	115 (36.74%)	894 (34.06%)	1.19 (0.93–1.52)	0.5266
GG	25 (7.99%)	135 (5.14%)	1.71 (1.08–2.69)	0.0473
Allele				
A	461 (73.64%)	4086 (77.83%)	Reference	
G	165 (26.36%)	1164 (22.17%)	1.26 (1.04–1.52)	0.0181
Dominant				
AA	173 (55.27%)	1596 (60.80%)	Reference	
AG/GG	140 (44.73%)	1029 (39.20%)	1.26 (0.99–1.59)	0.0589
Recessive				
AA/AG	288 (92.01%)	2490 (94.86%)	Reference	
GG	25 (7.99%)	135 (5.14%)	1.60 (1.03–2.50)	0.0377
rs724236				
Genotype				
AA	161 (51.44%)	1515 (57.74%)	Reference	
AT	121 (38.66%)	940 (35.82%)	1.21 (0.94–1.55)	0.5712
TT	31 (9.90%)	169 (6.44%)	1.73 (1.14–2.62)	0.0285
Allele				
A	443 (70.77%)	3970 (75.65%)	Reference	
T	183 (29.23%)	1278 (24.35%)	1.28 (1.07–1.54)	0.0077
Dominant				
AA	161 (51.44%)	1515 (57.74%)	Reference	
AT/TT	152 (48.56%)	1109 (42.26%)	1.29 (1.02–1.63)	0.0333
Recessive				
AA/AT	282 (90.10%)	2455 (93.56%)	Reference	
TT	31 (9.90%)	169 (6.44%)	1.60 (1.07–2.39)	0.0226

HPV16 and HPV18 were the two most carcinogenic HPV genotypes. Thus, we further analyzed the SNPs for HPV 16/18 susceptibility (Table 2) and found that TT genotype of rs2575712 in *SDC2* was associated with an increased HPV 16/18 susceptibility compared with GG genotype (TT versus GG, *OR *= 2.78, 95% *CI *= 1.22-6.36, *P* = 0.0278). This observation was consistent with the increased frequency of the allele T compared with G (T versus G, *OR *= 1.62, 95% *CI *= 1.11-2.37, *P* = 0.0119) and the dominant model (GT/TT versus GG, *OR *= 2.29, 95% *CI *= 1.08-4.85, *P* = 0.0313).

**TABLE 2:** Genotype and allele frequencies that were significant differences in patients with HPV16/18 (+) versus HPV (-)

SNP	HPV16/18 (+) (*n* = 57)	HPV (-) (*n* = 2625)	*OR* (95% *CI*)	*P* value
rs2575712				
Genotype				
GG	8 (14.04%)	713 (27.17%)	Reference	
GT	29 (50.88%)	1270 (48.40%)	2.04 (0.93–4.48)	0.4807
TT	20 (35.09%)	641 (24.43%)	2.78 (1.22–6.36)	0.0278
Allele				
G	45 (39.47%)	2696 (51.37%)	Reference	
T	69 (60.53%)	2552 (48.63%)	1.62 (1.11–2.37)	0.0119
Dominant				
GG	8 (14.04%)	713 (27.17%)	Reference	
GT/TT	49 (85.96)	1911 (72.83)	2.29 (1.08–4.85)	0.0313
Recessive				
GG/GT	37 (64.91%)	1983 (75.57%)	Reference	
TT	20 (35.09%)	641 (24.43%)	0.60 (0.35–1.04)	0.0647

### Associations between SNPs and persistent HPV infection.

Due to immune mechanisms, most HPV infections spontaneously cleared within one to 2 years (22). In the present study, the follow-up information on HPV was available in 202 patients with baseline HPV infection. 107 (52.97%) were classified as persistent HPV infection, while 95 (47.03%) HPV were spontaneously cleared, and the baseline characteristics of participants were listed in Table S4. Women younger than 45 were apt to spontaneously clear. Four SNPs (rs1047057 and rs10510097 in *FGFR2*, rs2575735 in *SDC2*, and rs878949 in *HSPG2*) were significantly associated with persistent HPV infection (Table S5). For rs1047057, HPV positive women carried AG genotype (AG versus GG, *OR *= 0.40, 95% *CI *= 0.21 to 0.74, *P* = 0.0159) and dominant model (AG/AA versus GG, *OR *= 0.45, 95% *CI *= 0.25 to 0.81, *P* = 0.0071) were prone to HPV cleared compared with GG genotype. For rs10510097, allele T increased the risk of persistent HPV infection (T versus C, *OR *= 1.74, 95% *CI *= 1.08 to 2.80, *P* = 0.0230) compared with C, while rs2575735 (T versus C, *OR *= 0.59, 95% *CI *= 0.36 to 0.98, *P* = 0.0404) and rs878949 (T versus C, *OR *= 0.52, 95% *CI *= 0.30 to 0.92, *P* = 0.0241) had the opposite tendency. Dominant model analysis of rs10510097 revealed that CT/TT was more likely to turn to persistent HPV infection compared to CC genotype (*OR *= 1.83, 95% *CI *= 1.03 to 3.22, *P* = 0.0373), while rs878949 (*OR *= 0.44, 95% *CI *= 0.23 to 0.83, *P* = 0.0099) had the opposite tendency.

### Associations between SNPs and disease progression.

The baseline characteristics of participants of current study between <CIN2 and CIN2+ groups were listed in Table S4. As expected, the proportion of HPV positive women in CIN2+ group was higher than <CIN2 group, and there were no differences in age, smoking status, alcohol drinking, and education between <CIN2 and CIN2+ groups. The frequencies of genotype and allele of two SNPs (rs16894821 in *SDC2*, and rs11199993 in *FGFR2*) were significantly associated with the disease progression (Table 3). For rs16894821, the genotype homozygotes GG was more frequent in the CIN2+ group, and the homozygotes AA was more frequent in the <CIN2 group. The carriers of GG genotype of rs16894821 would increase the risk of CIN2+ compared with AA (*OR *= 2.57, 95% *CI *= 1.16 to 5.68, *P* = 0.0274). Recessive model analysis revealed that genotype GG also increased the risk of disease progression compared with AA/AG (*OR *= 2.40, 95% *CI *= 1.12 to 5.15, *P* = 0.0241). For rs11199993, dominant model of rs11199993 carried CG/CC genotype (versus GG, *OR *= 1.64, 95% *CI *= 1.01 to 2.68, *P* = 0.0468) and allele C (versus G, *OR *= 1.52, 95% *CI *= 1.03 to 2.22, *P* = 0.0330) were also risk factors for developing to CIN2+.

**TABLE 3:** Genotype and allele frequencies that were significant differences between <CIN2 and CIN2+ groups

SNP	<CIN2 (*n* = 2193)	CIN2+ (*n* = 66)	*OR* (95% *CI*)	*P* value
rs16894821				
Genotype				
AA	1299 (59.23%)	34 (51.52%)	Reference	
AG	775 (35.34%)	24 (36.36%)	1.18 (0.70–2.01)	0.2946
GG	119 (5.43%)	8 (12.12%)	2.57 (1.16–5.68)	0.0274
Allele				
A	3373 (76.90%)	92 (69.70%)	Reference	
G	1013 (23.10%)	40 (30.30%)	1.45 (0.99–2.11)	0.0549
Dominant				
AA	1299 (59.23%)	34 (51.52%)	Reference	
AG/GG	894 (40.77%)	32 (48.48%)	1.37 (0.84–2.23)	0.2106
Recessive				
AA/AG	2074 (94.57%)	58 (87.88%)	Reference	
GG	119 (5.43%)	8 (12.12%)	2.40 (1.12–5.15)	0.0241
rs11199993				
Genotype				
GG	1363 (62.18%)	33 (50.00%)	Reference	
CG	736 (33.58%)	28 (42.42%)	2.22 (0.85–5.82)	0.2308
CC	93 (4.24%)	5 (7.58%)	1.57 (0.94–2.62)	0.8653
Allele				
G	3462 (78.97%)	94 (71.21%)	Reference	
C	922 (21.03%)	38 (28.79%)	1.52 (1.03–2.22)	0.0330
Dominant				
GG	1363 (62.18%)	33 (50.00%)	Reference	
CG/CC	829 (37.82%)	33 (50.00%)	1.64 (1.01–2.68)	0.0468
Recessive				
GG/CG	2099 (95.76%)	61 (92.42%)	Reference	
CC	93 (4.24%)	5 (7.58%)	1.85 (0.73–4.71)	0.1972

### The performance of triage strategy based on SNP in detecting CIN2+.

Recessive model of rs16894821 and dominant model of rs11199993 showed the potential for triaging women with HPV infection. Next, we evaluated the efficacy of SNP’s triage strategy and found that SNP has comparable screening efficacy with cervical cytology in identifying CIN2+ in women with non-16/18 HPV infection. The sensitivities were 0.51 (0.36 to 0.66) and 0.44 (0.30 to 0.60), specificities were 0.96 (0.96 to 0.97) and 0.98 (0.97 to 0.99), positive predictive values (PPVs) were 0.23 (0.15 to 0.33) and 0.33 (0.22 to 0.47), and negative predictive values (NPVs) were 0.99 (0.98 to 0.99) and 0.99 (0.98 to 0.99) for SNPs and cytology, respectively (Table 4), which indicated that SNP could effectively identify the disease progression in women with non-HPV16/18 infection.

**TABLE 4:** The efficacy of detecting the CIN2+ in patients with non-HPV16/18 positive based on cytology and SNPs^*a*^

Strategies	Sensitivity (95% *CI*)	Specificity (95% *CI*)	PPV (95% *CI*)	NPV (95% *CI*)
Cytology	0.44 (0.30–0.60)	0.98 (0.97–0.99)	0.33 (0.22–0.47)	0.99 (0.98–0.99)
SNPs	0.51 (0.36–0.66)	0.96 (0.96–0.97)	0.23 (0.15–0.33)	0.99 (0.98–0.99)

a PPV, positive predictive value; NPV, negative predictive value.

## DISCUSSION

HPV infection was recognized as the principal factor in the development of CC. More than 80% of women who in sexually active might be infected by the virus, but only a very few persist and eventually cause cancer (23). In practice, once a woman was identified as HPV positive, it would cause anxiety and panic to them. The outcome of HPV infection is multifactorial and related to many genetic and nongenetic factors. The current study evaluated the association of SNPs in the HPV receptor associated genes with the natural process of HPV infection and CIN2+ risk based on a longitudinal study of Chinese population. Our evidence indicated that genetic variants played an important role in predicting HPV susceptibility and clinical outcomes. In addition, we found that SNPs might be a potential biomarker for identifying CIN2+ in patients with non-16/18 HPV infection, which could make up for the lack of cytologists in developing countries.

*SDC2* was one of the syndecan family of proteins, which could promote cell adhesion and was associated with cell proliferation. Previous studies have reported that *SDC2* was overexpressed and enhanced invasion in several cancers (24, 25). Decreasing *SDC2* expression led to cell cycle arrest and reduced the occurrence of cancers, such as colon and breast cancer (26, 27). In addition, Oh et al. identified that methylated *SDC2* could be used as a serum DNA biomarker for the early detection of colon cancer (28). In current study, we also found that SNP sites rs16894821 and rs724236 in *SDC2* gene were associated with the susceptibility to HPV, and rs16894821 was related with the cervical disease. Rs2575712 in *SDC2* gene significantly increased HPV16/18 susceptibility, which was consistent with our previous study (12). These results indicated that genetic variants of *SDC2* could reflect the susceptibility of HPV.

High-risk HPV was found in over 95% of CC patients (29–31) and persistent infection was associated with an increased risk of precancerous lesions or invasive carcinomas. *HSPG2* was a large multidomain extracellular matrix proteoglycan. Zhang et al. reported that *HSPG2* was lower expressed in CC patients compared with healthy people (32). In solid tumors, high expression of *HSPG2* always indicated the disease invasion, metastasis, and angiogenesis of solid tumors (33, 34). Our results revealed rs2575735 in *SDC2* gene and rs878949 in *HSPG2* gene were both related to persistent HPV infection. These results indicated that genetic variants of *SDC2* and *HSPG2* genes could predict the outcomes of women with HPV infection.

As we know, women with HPV16/18 infection would be directly referred to colposcopy, while women with non-16/18 HPV infection would receive cervical cytology triage, and abnormal results (atypical squamous cells with unknown significance [ASC-US] or worse, ≥ ASC-US) required further colposcopy. However, the performance of cytology was heavily dependent on the experience and abilities of trained cytologists. In addition, a lack of well-trained cytologists had limited the application of cytology in developing countries. *FGFR2* gene, located on human chromosome 10q26, encoded *FGFR2b* and *FGFR2c* isoforms functioning as *FGF* receptors (35), which played an important role in tumorigenesis by regulating cell proliferation, apoptosis, metastasis, and angiogenesis (36). Increasing evidence reported that *FGFR2* was associated with cancers, such as endometrial uterus cancer, gastric cancer, and ovarian cancer (37–39). Studies on CC revealed that *FGFR2* was highly expressed in CC tissues and cell lines (40). Sun et al. revealed that *FGFR2* was a direct and functional downstream target of miR-889 in CC cells, and miR-889 overexpression suppressed CC viability and invasion by targeting *FGFR* (41), and *FGFR2* has become a new target for female reproductive system cancers therapy (42). In present study, we found that genotype CG/CC of rs11199993 in *FGFR2* gene was more frequent in the CIN2+ group compared to GG. Importantly, our results demonstrated that SNPs (rs16894821 using the recessive model and rs11199993 using the dominant model) had a similar effect in detecting disease progression as cervical cytology in women with non-HPV16/18 infection. To the best of our knowledge, our study was the first to provide evidence that SNPs of HPV host cell receptor gene can be used to identify the disease progression in women with non-HPV16/18 infection.

Understanding the role of genetic variants could promote the development of early detection biomarkers, predict the outcome of HPV infection and disease progression, and make aggressive management to reduce the incidence of adverse outcomes in a high-risk population. Our findings suggested that SNPs could effectively identify CIN2+ in women with non-HPV16/18 infection and had comparable PPV and NPV with cytology triage strategy. Due to the small sample size, additional prospective large-sample studies are needed to address the functional consequence of this genetic alteration in the natural course of HPV infection.

## MATERIALS AND METHODS

### Study population.

Between October 2016 and March 2020, 3,066 women were enrolled in a 3-year prospective clinical trial on CC screening in Lishui, Zhejiang, China approved by the National Medical Products Administration clinical trial (Approval number: 20160149, Shanghai). Eligibility criteria for clinical trial included: aged 21 to 65 years and informed consent were obtained. Exclusion criteria included as follows: pregnancy or within 2 months of the postpartum period; previous total hysterectomy; surgical history of cervix uteri; a history of CIN or worse, vulvar intraepithelial neoplasia or worse, or vaginal intraepithelial neoplasia or worse, invalid HPV results/invalid or unsatisfactory cytology. Women with insufficient residual cytology samples were further excluded from current study. This study was approved by the Ethics Committee of Women’s Hospital, School of Medicine, Zhejiang University (approval number: IRB-20220035-R).

### Screening protocol of clinical trials.

**FIG 1:**
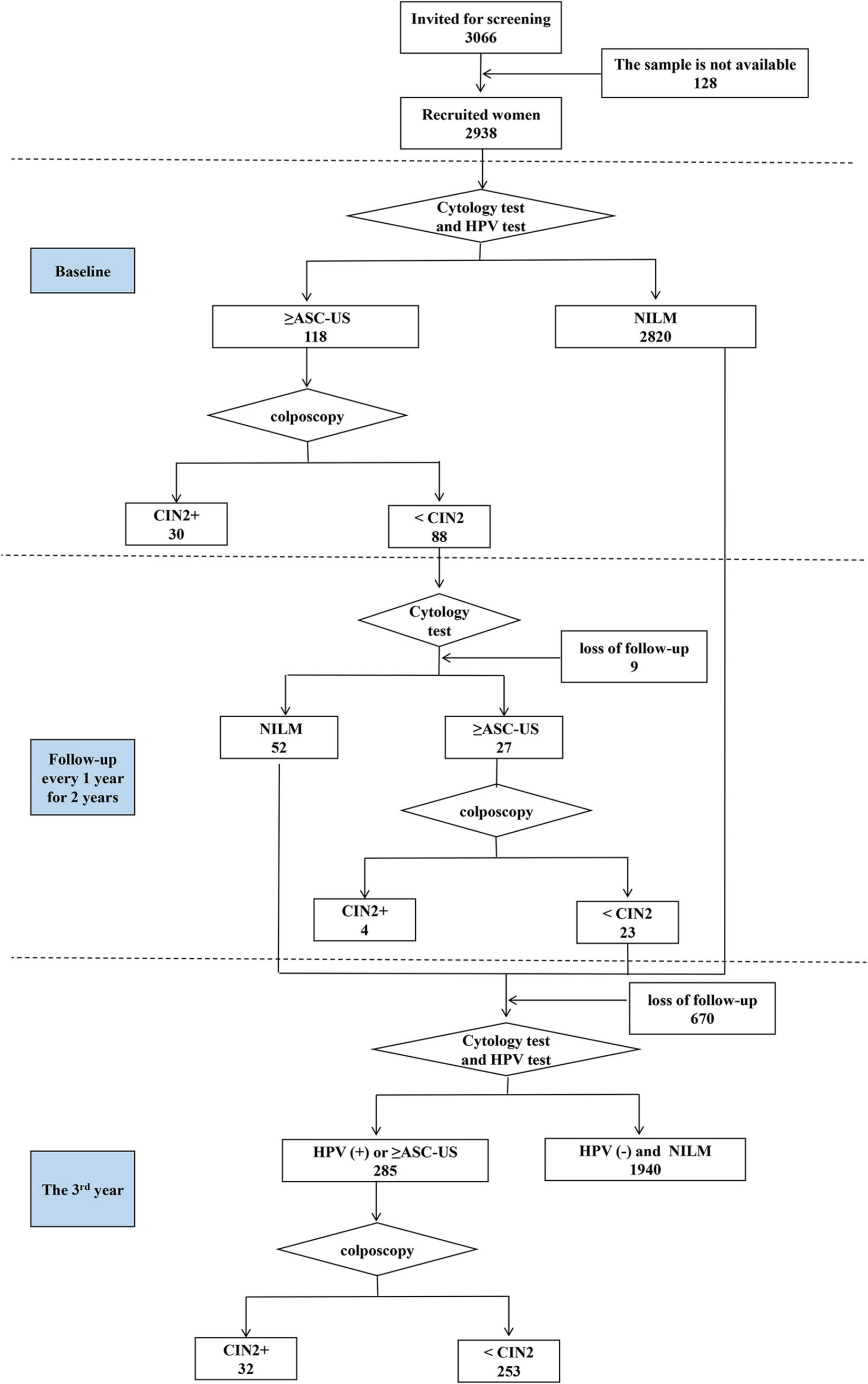
The flow chart of current study.

The flow chart was shown in Fig. 1. At baseline, cervical exfoliated cells were collected using a cytology brush (Hologic, Bedford, MA) and stored in the tubes with preservation solution for the cytology test (Hologic, Bedford, MA) and HPV test, respectively. HPV was detected by Cobas 4800 Amplification/Detection kit (Roche Molecular Systems, Pleasanton, CA) according to the manufacturer’s instruction, which was featured with automated sample preparation combined with real-time PCR technology for detecting 14 high-risk HPV genotypes. Women with positive HPV 16/18 or abnormal cytology results (≥ ASC-US) were referred to colposcopy with or without biopsy specimen. Due to clinical experience and ethical consideration, women with both negative HPV and cytology results were not referred to colposcopy and were regarded as histological low-grade squamous intraepithelial lesion or less by default. The pathological diagnostic standard was based on the World Health Organization Classification of Tumors of the Female Genital Tract (43).

Subsequently, women with positive HPV or abnormal cytology at baseline would be recalled for cytology test every 1 year for 2 years and all enrolled women would be recalled for cytology and HPV test at 3rd year. Women with positive HPV or ≥ASC-US were referred to colposcopy in follow-up. All women with histological abnormalities at each visit were managed according to the Guideline for Comprehensive Prevention and Control of Cervical Cancer in China (44). The endpoint was CIN2+. Women who had HPV infection from baseline to the 3rd year of follow-up were defined as having persistent HPV infection.

### DNA extraction and SNP detection.

Genomic DNA was extracted using the AxyPrep Multisource Genomic DNAMinprep kit (Axygen, Hangzhou, China). The genotypes of 29 SNP sites of HPV receptor related genes, including EGFR, FGFR2, HSPG2, INTEGRIN, and SDC2, were determined using the MassARRAY System (Sequenom Inc., San Diego, CA, USA) at Sangon Biotech Company (Shanghai, China). The detection system was based on MALDI-TOF MS, and the following procedures were applied: PCR amplification of genomic DNA containing the SNP sites, dNTP degradation using shrimp alkaline phosphatase (SAP), single base extension, desalinization with Spectro CLEAN resin, sample dispensation on Nano dispenser Spectro CHIP, data acquisition and analysis with MassARRAY TYPER software. Primers used for PCR amplification, single base extension, and 29 SNP sites were listed in Table S1. The detection of 29 SNP sites for each sample was carried out simultaneously in one well.

### Statistical analysis.

Categorical variables were expressed as absolute numbers with percentages and compared with the χ^2^ test. The χ^2^ or Fisher exact tests were utilized to analyze the differences in genotypic and allelic distributions between groups. The correlation of genotypic and allelic with the risk of CIN2+ was evaluated via *OR* and 95% *CI* by unconditional logistic regression. The *HWE* was estimated by the goodness-of-χ*^2^* test. All statistical tests were two-sided, and *P* < 0.05 was considered statistically significant. We used SAS 9.4 for all statistical analyses.
