# Supplementary material for: The Significance of Human Papillomavirus Receptors Related Genetic Variants in Cervical Cancer Screening
Source: Microbiol Spectr. 2023 Jun 26;11(4):e05117-22. doi: 10.1128/spectrum.05117-22 (PMC10434196; doi:10.1128/spectrum.05117-22)
Supplement: Supplemental file 1 — Supplemental material. Download spectrum.05117-22-s0001.pdf, PDF file, 0.6 MB [file spectrum.05117-22-s0001.pdf]

Supplementary data

Table S1. 29 SNPs and primers used for PCR amplification and single base extension

gene_symbol	SNP_ID	PCR primer 1	PCR primer 2	Extended primer direction	Single base extension
EGFR	rs3778866*	ACGTTGGATGCCAGGTTTGCCTGTTCTTAC	ACGTTGGATGGTACTGTATGCATATTGTTG	R	gATTGCCGGAGTAACTT
EGFR	rs6956366*	ACGTTGGATGTCAGGTGACATCCCACCAAC	ACGTTGGATGAAAACTGGCCCAGGACTATG	F	gTCCCACCAACCCTCACTTT
EGFR	rs11770506*	ACGTTGGATGTGCTCTTGCTTCCAGATTGC	ACGTTGGATGTCTTGTATTCTGTGCTACGG	F	ggagtACACCAGTGGTTTGTG
EGFR	rs4947972	ACGTTGGATGTGTTCTGATTTCCGTGTTTG	ACGTTGGATGGATTCCAAAACGAGGTAGAG	F	ggacTGGAATTGCTTTTGTGTT
EGFR	rs12718946	ACGTTGGATGCACCGCTATAATGTGTGAAC	ACGTTGGATGTTTCTTAGGTGCACTGTCCC	F	TCCATCATCTATACGTTAGTAAA
EGFR	rs4947974*	ACGTTGGATGGAGGACTTAGGTCCCAAAGC	ACGTTGGATGAGACTGGAAACTCGAGGAAG	R	ggagTAGGTCCCAAAGCCCAGTGAC

EGFR	rs28384376	ACGTTGGATGGAAAACAA CACCCTGGTCTG	ACGTTGGATGCTTCACTT TCCACTCACCCG	F	ggtggGCCACCTGTGCCATCCAAACT
EGFR	rs12668421*	ACGTTGGATGTAAAAACG TGGAGTGAGCCC	ACGTTGGATGTTTATCTG GTGTCCACTCTG	F	ggggcGGTGCCATTTATCCAGCCCATAC
EGFR	rs11977660*	ACGTTGGATGCAAAGTG GATCTGTGATGTC	ACGTTGGATGCCAGCAA CTTCCTTTCTAAC	R	caggCTTGGAGTATCCAGCATATGTGAA
FGFR2	rs2981427*	ACGTTGGATGGTTAACTA GTTCTTGCCTTC	ACGTTGGATGATCCCTCG AACAAATGGGTA	R	TTGCCTTCCCTAGGT
FGFR2	rs2981432*	ACGTTGGATGTGATTTCA GTCTCCTGTGGG	ACGTTGGATGTGAGACA GCACCAAATCAGG	R	CCTGTGGGAGATTTGT
FGFR2	rs11199993*	ACGTTGGATGAGGCCAT AGAGTTAGCAAGG	ACGTTGGATGGGTGGAA ATTACGTGGAGAG	F	AGGGTGGAAACAGAACTT
FGFR2	rs1047057	ACGTTGGATGTAACAAGG AAGGCAGAACGC	ACGTTGGATGCATGTAAC CCCTCTCACCTG	F	tccgACTGGTCCACAGCCA
FGFR2	rs3135761*	ACGTTGGATGCCGACAT CTCTATTCTAGTG	ACGTTGGATGAGCTAGC AAACCACAGTCTC	R	ccttCATCTGCCTCTTTGTCATTT
FGFR2	rs2936870*	ACGTTGGATGTCCAGTTT AAACACGACCCC	ACGTTGGATGCCACAGC AATCCAATATGAG	F	ctACCCCAGTTATTTCATAGCCATG

FGFR2	rs10510097	ACGTTGGATGGCTGATCA TGTGATGTTGTC	ACGTTGGATGAACAGCA CCCACCATGTTAG	R	caccGATCATTTTATGACCCAGACT
HSPG2	rs6697265	ACGTTGGATGCTGGAAT GCCCTTTCCATTG	ACGTTGGATGTGAAGGA GACCTGAAGTAAG	F	GCCCTTTCCATTGATATCTGC
HSPG2	rs6680566	ACGTTGGATGATGGGAA CGGAGTCTGAAAC	ACGTTGGATGCCTCTGA GCTAGCCAAATAC	F	CCCCTCTGCTGGGTTCA
HSPG2	rs878949	ACGTTGGATGTCTCCGTA TCTGTACCTCTC	ACGTTGGATGGAAAGGA CTAAGAACGGAGG	R	ggagGTGCCCCACTCCTGT
HSPG2	rs12034979	ACGTTGGATGAGCAGTTT AAGGAGACAGCC	ACGTTGGATGTATCCATA TGACCCTCTCCC	F	ctttgAGGAGACAGCCATTATGGGAAGA
ITGA6	rs16860426	ACGTTGGATGCTCGGGG AAGGTTTTTAAGC	ACGTTGGATGCAGGACC TTTGCACAGTTTC	F	tgaatGGGTGAGTAGGAGGATGCCTG
SDC2	rs880938*	ACGTTGGATGCAGAGTA CCGCAGCGATTG	ACGTTGGATGAGCAGGC GCAGGAGGAGGAA	R	AGCGATTGCGGCTCA
SDC2	rs1126681*	ACGTTGGATGAGAAGCTT CAGGAGTGTATC	ACGTTGGATGTTAGAAGC AGCCACCTTACC	F	CCTATTGATGACGATGACTA
SDC2	rs2575712	ACGTTGGATGCCCAGTT GTACTGGTAAAAG	ACGTTGGATGGGTGGTT GACAATTACACTG	F	TGTAGGAATCATTCCTTTGTC

SDC2	rs2575738	ACGTTGGATGTGTCCTCA CCCTTAAGCAG	ACGTTGGATGCAAAGATC ACATGACTAGGC	F	ctCCTTAAGCAGCTGATTTTTC
SDC2	rs2575735	ACGTTGGATGTCTGGCT GGAGAAAAGTTAG	ACGTTGGATGCCTTCTTT TTCCGAAGTAGC	F	ctaggTAGAACAGAAGGGAACGC
SDC2	rs724236*	ACGTTGGATGTCTGCCTC CATGAACTTGAC	ACGTTGGATGTACGTTGG GTTAAATAGAGC	R	aCAGTTACCTCATAAGTGCAATAA
SDC2	rs16894821	ACGTTGGATGAACCCAA GCGGTGTTTACTG	ACGTTGGATGCATCAACT GCCTCCTCTTAG	F	acttGGGGAGGGAAGCCTTGCCTTTA
SDC2	rs999681*	ACGTTGGATGTGCTAAAA CCACTAGCCATC	ACGTTGGATGGGGCTGA AAAGATTGTACTG	R	ggagCTTTCATTTTCTGTACCATTTATA

* Tag SNPs

Table S2. Baseline characteristics of participants of the Lishui cohort

	N (%)
Age (years)	
<45	1269(43.19%)
≥45	1669(56.81%)
Smoking status	
Non-smoke	2931(99.76%)
Smoke	7(0.24%)
Alcohol drinking	
Never drink	2269(77.23%)
Drink	669(22.77%)
Education	
<Junior school	1799(61.23%)
≥Junior school	1139(38.77%)
Reproductive age (year)	
<24	1221(41.56%)
24-29	1553(52.86%)
>29	164(5.58%)

Table S3. SNPs genotypes and their allelic distribution between groups with and without HPV infection

	HPV+ (n=313)	HPV- (n=2625)
**rs1047057**		
Genotype		
AA	49(15.65%)	438(16.69%)
AG	163(52.08%)	1307(49.79%)
GG	101(32.27%)	880(33.52%)
Allele		
A	261(41.69%)	2183(41.58%)
G	365(58.31%)	3067(58.42%)
Dominant		
GG	101(32.27%)	880(33.52%)
AG/AA	212(67.73%)	1745(66.48%)
Recessive		
GG/AG	264(84.35%)	2187(83.31%)
AA	49(15.65%)	438(16.69%)
**rs10510097**		
Genotype		
CC	181(58.01%)	1638(62.40%)
CT	121(38.78%)	850(32.38%)
TT	10(3.21%)	137(5.22%)
Allele		
C	483(77.40%)	4126(78.59%)
T	141(22.60%)	1124(21.41%)
Dominant		
CC	181(58.01%)	1638(62.40%)
CT/TT	131(41.99%)	987(37.60%)
Recessive		
CC/CT	302(96.79%)	2488(94.78%)
TT	10(3.21%)	137(5.22%)
**rs11199993**		
Genotype		
CC	16(5.11%)	118(4.50%)
CG	109(34.82%)	878(33.47%)
GG	188(60.06%)	1627(62.03%)

Allele		
C	141(22.52%)	1114(21.24%)
G	485(77.48%)	4132(78.76%)
Dominant		
GG	188(60.06%)	1627(62.03%)
CG/CC	125(39.94%)	996(37.97%)
Recessive		
GG/CG	297(94.89%)	2505(95.50%)
CC	16(5.11%)	118(4.50%)
**rs1126681**		
Genotype		
CC	127(40.58%)	1056(40.27%)
CT	149(47.60%)	1232(46.99%)
TT	37(11.82%)	334(12.74%)
Allele		
C	403(64.38%)	3344(63.77%)
T	223(35.62%)	1900(36.23%)
Dominant		
CC	127(40.58%)	1056(40.27%)
CT/TT	186(59.42%)	1566(59.73%)
Recessive		
CC/CT	276(88.18%)	2288(87.26%)
TT	37(11.82%)	334(12.74%)
**rs11770506**		
Genotype		
CC	135(43.13%)	1087(41.44%)
CT	142(45.37%)	1226(46.74%)
TT	36(11.50%)	310(11.82%)
Allele		
C	412(65.81%)	3400(64.81%)
T	214(34.19%)	1846(35.19%)
Dominant		
CC	135(43.13%)	1087(41.44%)
CT/TT	178(56.87%)	1536(58.56%)
Recessive		
CC/CT	277(88.50%)	2313(88.18%)
TT	36(11.50%)	310(11.82%)
**rs11977660**		

Genotype		
CC	139(44.41%)	1086(41.37%)
CT	137(43.77%)	1200(45.71%)
TT	37(11.82%)	339(12.91%)
Allele		
C	415(66.29%)	3372(64.23%)
T	211(33.71%)	1878(35.77%)
Dominant		
CC	139(44.41%)	1086(41.37%)
CT/TT	174(55.59%)	1539(58.63%)
Recessive		
CC/CT	276(88.18%)	2286(87.09%)
TT	37(11.82%)	339(12.91%)
**rs12034979**		
Genotype		
AA	1(0.32%)	0(0.00%)
AG	28(8.95%)	192(7.32%)
GG	284(90.73%)	2431(92.68%)
Allele		
A	30(4.79%)	192(3.66%)
G	596(95.21%)	5054(96.34%)
Dominant		
GG	284(90.73%)	2431(92.68%)
AG/AA	29(9.27%)	192(7.32%)
Recessive		
GG/AG	312(99.68%)	2623(100.00%)
AA	1(0.32%)	0(0.00%)
**rs12668421**		
Genotype		
AA	11(3.51%)	90(3.43%)
AT	85(27.16%)	766(29.19%)
TT	217(69.33%)	1768(67.38%)
Allele		
A	107(17.09%)	946(18.03%)
T	519(82.91%)	4302(81.97%)

Dominant		
TT	217(69.33%)	1768(67.38%)
AT/AA	96(30.67%)	856(32.62%)
Recessive		
TT/AT	302(96.49%)	2534(96.57%)
AA	11(3.51%)	90(3.43%)
**rs12718946**		
Genotype		
CC	131(41.85%)	976(37.21%)
CG	144(46.01%)	1270(48.42%)
GG	38(12.14%)	377(14.37%)
Allele		
C	406(64.86%)	3222(61.42%)
G	220(35.14%)	2024(38.58%)
Dominant		
CC	131(41.85%)	976(37.21%)
CG/GG	182(58.15%)	1647(62.79%)
Recessive		
CC/CG	275(87.86%)	2246(85.63%)
GG	38(12.14%)	377(14.37%)
**rs16860426**		
Genotype		
AA	164(52.40%)	1364(51.98%)
AT	122(38.98%)	1039(39.60%)
TT	27(8.63%)	221(8.42%)
Allele		
A	450(71.88%)	3767(71.78%)
T	176(28.12%)	1481(28.22%)
Dominant		
AA	164(52.40%)	1364(51.98%)
AT/TT	149(47.60%)	1260(48.02%)
Recessive		
AA/AT	286(91.37%)	2403(91.58%)
TT	27(8.63%)	221(8.42%)
**rs16894821**		
Genotype		

AA	173(55.27%)	1596(60.80%)
AG	115(36.74%)	894(34.06%)
GG	25(7.99%)	135(5.14%)
Allele		
A	461(73.64%)	4086(77.83%)
G	165(26.36%)	1164(22.17%)
Dominant		
AA	173(55.27%)	1596(60.80%)
AG/GG	140(44.73%)	1029(39.20%)
Recessive		
AA/AG	288(92.01%)	2490(94.86%)
GG	25(7.99%)	135(5.14%)
**rs2575712**		
Genotype		
GG	75(24.04%)	713(27.17%)
GT	153(49.04%)	1270(48.40%)
TT	84(26.92%)	641(24.43%)
Allele		
G	303(48.56%)	2696(51.37%)
T	321(51.44%)	2552(48.63%)
Dominant		
GG	75(24.04%)	713(27.17%)
GT/TT	237(75.96%)	1911(72.83%)
Recessive		
GG/GT	228(73.08%)	1983(75.57%)
TT	84(26.92%)	641(24.43%)
**rs2575735**		
Genotype		
CC	205(65.50%)	1655(63.05%)
CT	92(29.39%)	861(32.80%)
TT	16(5.11%)	109(4.15%)
Allele		
C	502(80.19%)	4171(79.45%)
T	124(19.81%)	1079(20.55%)
Dominant		
CC	205(65.50%)	1655(63.05%)
CT/TT	108(34.50%)	970(36.95%)

Recessive		
CC/CT	297(94.89%)	2516(95.85%)
TT	16(5.11%)	109(4.15%)
**rs2575738**		
Genotype		
AA	23(7.35%)	171(6.52%)
AG	111(35.46%)	996(37.96%)
GG	179(57.19%)	1457(55.53%)
Allele		
A	157(25.08%)	1338(25.50%)
G	469(74.92%)	3910(74.50%)
Dominant		
GG	179(57.19%)	1457(55.53%)
AG/AA	134(42.81%)	1167(44.47%)
Recessive		
GG/AG	290(92.65%)	2453(93.48%)
AA	23(7.35%)	171(6.52%)
**rs28384376**		
Genotype		
GG	313(100.00%)	2625(100.00%)
Allele		
G	626(100.00%)	5250(100.00%)
**rs2936870**		
Genotype		
CC	128(40.89%)	1024(39.02%)
CT	140(44.73%)	1234(47.03%)
TT	45(14.38%)	366(13.95%)
Allele		
C	396(63.26%)	3282(62.54%)
T	230(36.74%)	1966(37.46%)
Dominant		
CC	128(40.89%)	1024(39.02%)
CT/TT	185(59.11%)	1600(60.98%)
Recessive		
CC/CT	268(85.62%)	2258(86.05%)
TT	45(14.38%)	366(13.95%)
**rs2981427**		

Genotype		
CC	66(21.09%)	494(18.83%)
CT	147(46.96%)	1264(48.17%)
TT	100(31.95%)	866(33.00%)
Allele		
C	279(44.57%)	2252(42.91%)
T	347(55.43%)	2996(57.09%)
Dominant		
TT	100(31.95%)	866(33.00%)
CT/CC	213(68.05%)	1758(67.00%)
Recessive		
TT/CT	247(78.91%)	2130(81.17%)
CC	66(21.09%)	494(18.83%)
**rs2981432**		
Genotype		
AA	110(35.48%)	890(33.94%)
AG	146(47.10%)	1252(47.75%)
GG	54(17.42%)	480(18.31%)
Allele		
A	366(59.03%)	3032(57.82%)
G	254(40.97%)	2212(42.18%)
Dominant		
AA	110(35.48%)	890(33.94%)
AG/GG	200(64.52%)	1732(66.06%)
Recessive		
AA/AG	256(82.58%)	2142(81.69%)
GG	54(17.42%)	480(18.31%)
**rs3135761**		
Genotype		
AA	78(24.92%)	700(26.67%)
AG	170(54.31%)	1313(50.02%)
GG	65(20.77%)	612(23.31%)
Allele		
A	326(52.08%)	2713(51.68%)
G	300(47.92%)	2537(48.32%)
Dominant		
AA	78(24.92%)	700(26.67%)

AG/GG	235(75.08%)	1925(73.33%)
Recessive		
AA/AG	248(79.23%)	2013(76.69%)
GG	65(20.77%)	612(23.31%)
**rs3778866**		
Genotype		
AA	141(45.48%)	1103(42.37%)
AC	142(45.81%)	1207(46.37%)
CC	27(8.71%)	293(11.26%)
Allele		
A	424(68.39%)	3413(65.56%)
C	196(31.61%)	1793(34.44%)
Dominant		
AA	141(45.48%)	1103(42.37%)
AC/CC	169(54.52%)	1500(57.63%)
Recessive		
AA/AC	283(91.29%)	2310(88.74%)
CC	27(8.71%)	293(11.26%)
**rs4947972**		
Genotype		
CC	226(72.20%)	1918(73.09%)
CG	80(25.56%)	653(24.89%)
GG	7(2.24%)	53(2.02%)
Allele		
C	532(84.98%)	4489(85.54%)
G	94(15.02%)	759(14.46%)
Dominant		
CC	226(72.20%)	1918(73.09%)
CG/GG	87(27.80%)	706(26.91%)
Recessive		
CC/CG	306(97.76%)	2571(97.98%)
GG	7(2.24%)	53(2.02%)
**rs4947974**		
Genotype		
CC	28(8.97%)	282(10.75%)
CT	137(43.91%)	1195(45.54%)
TT	147(47.12%)	1147(43.71%)
Allele		

C	193(30.93%)	1759(33.52%)
T	431(69.07%)	3489(66.48%)
Dominant		
TT	147(47.12%)	1147(43.71%)
CT/CC	165(52.88%)	1477(56.29%)
Recessive		
TT/CT	284(91.03%)	2342(89.25%)
CC	28(8.97%)	282(10.75%)
**rs6680566**		
Genotype		
CC	51(16.29%)	412(15.71%)
CT	144(46.01%)	1263(48.15%)
TT	118(37.70%)	948(36.14%)
Allele		
C	246(39.30%)	2087(39.78%)
T	380(60.70%)	3159(60.22%)
Dominant		
TT	118(37.70%)	948(36.14%)
CT/CC	195(62.30%)	1675(63.86%)
Recessive		
TT/CT	262(83.71%)	2211(84.29%)
CC	51(16.29%)	412(15.71%)
**rs6697265**		
Genotype		
CC	69(22.04%)	548(20.88%)
CG	149(47.60%)	1339(51.01%)
GG	95(30.35%)	738(28.11%)
Allele		
C	287(45.85%)	2435(46.38%)
G	339(54.15%)	2815(53.62%)
Dominant		
GG	95(30.35%)	738(28.11%)
CG/CC	218(69.65%)	1887(71.89%)
Recessive		
GG/CG	244(77.96%)	2077(79.12%)
CC	69(22.04%)	548(20.88%)
**rs6956366**		
Genotype		

CC	225(71.88%)	1831(69.75%)
CG	76(24.28%)	729(27.77%)
GG	12(3.83%)	65(2.48%)
Allele		
C	526(84.03%)	4391(83.64%)
G	100(15.97%)	859(16.36%)
Dominant		
CC	225(71.88%)	1831(69.75%)
CG/GG	88(28.12%)	794(30.25%)
Recessive		
CC/CG	301(96.17%)	2560(97.52%)
GG	12(3.83%)	65(2.48%)
**rs724236**		
Genotype		
AA	161(51.44%)	1515(57.74%)
AT	121(38.66%)	940(35.82%)
TT	31(9.90%)	169(6.44%)
Allele		
A	443(70.77%)	3970(75.65%)
T	183(29.23%)	1278(24.35%)
Dominant		
AA	161(51.44%)	1515(57.74%)
AT/TT	152(48.56%)	1109(42.26%)
Recessive		
AA/AT	282(90.10%)	2455(93.56%)
TT	31(9.90%)	169(6.44%)
**rs878949**		
Genotype		
CC	230(73.48%)	1949(74.25%)
CT	78(24.92%)	631(24.04%)
TT	5(1.60%)	45(1.71%)
Allele		
C	538(85.94%)	4529(86.27%)
T	88(14.06%)	721(13.73%)
Dominant		
CC	230(73.48%)	1949(74.25%)
CT/TT	83(26.52%)	676(25.75%)
Recessive		
CC/CT	308(98.40%)	2580(98.29%)

TT	5(1.60%)	45(1.71%)
**rs880938**		
Genotype		
CC	88(28.21%)	779(29.70%)
CG	149(47.76%)	1304(49.71%)
GG	75(24.04%)	540(20.59%)
Allele		
C	325(52.08%)	2862(54.56%)
G	299(47.92%)	2384(45.44%)
Dominant		
CC	779(29.70%)	88(28.21%)
CG/GG	1844(70.30%)	224(71.79%)
Recessive		
CC/CG	237(75.96%)	2083(79.41%)
GG	75(24.04%)	540(20.59%)
**rs999681**		
Genotype		
GG	61(19.55%)	522(19.89%)
GT	143(45.83%)	1296(49.39%)
TT	108(34.62%)	806(30.72%)
Allele		
G	265(42.47%)	2340(44.59%)
T	359(57.53%)	2908(55.41%)
Dominant		
TT	108(34.62%)	806(30.72%)
GT/GG	204(65.38%)	1818(69.28%)
Recessive		
TT/GT	251(80.45%)	2102(80.11%)
GG	61(19.55%)	522(19.89%)

Table S4. The demographic characteristics associated with HPV persistence and cervical disease

	Outcome of HPV infection			Cervical disease		
	HPV persistent (n=95)	Spontaneously cleared (n=107)	*P*-value	<CIN2 (n=2193)	CIN2+ (n=66)	*P*-value
Age (years)						
<45	20(21.05%)	45(42.06%)	0.0013	897(40.90%)	31(46.97%)	0.3236
≥45	75(78.95%)	62(57.94%)		1296(59.10%)	35(53.03%)	
Smoking status						
Non-smoke	95(100.00%)	107(100.00%)	1.0000	2187(99.73%)	66(100.00%)	1.0000
Smoke	0(0.00%)	0(0.00%)		6(0.27%)	0(0.00%)	
Alcohol drinking						
Never drink	72(75.79%)	83(77.57%)	0.7651	1693(77.20%)	53(80.30%)	0.5533
Drink	23(24.21%)	24(22.43%)		500(22.80%)	13(19.70%)	
Education						
<Junior school	71(74.74%)	67(62.62%)	0.0634	1351(61.61%)	41(62.12%)	0.9323
≥Junior school	24(25.26%)	40(37.38%)		842(38.39%)	25(37.88%)	
HPV types						
HPV negative	/	/	0.9005	1965(89.60%)	6(9.09%)	<0.0001
HPV 16/18	13(13.68%)	14(13.08%)		32(1.46%)	21(31.82%)	
HPV non-16/18	82(86.32%)	93(86.92%)		196(8.94%)	39(59.09%)	

Table S5. Genotype and allele frequencies of SNPs that significantly associated with the outcomes in patients with HPV infection

	HPV persistent (n=95)	Spontaneously cleared (n=107)	*OR (95% CI)*	*P*-value
rs1047057				
Genotype				
GG	41(43.16%)	27(25.23%)	Reference	
AG	36(37.89%)	61(57.01%)	0.40(0.21- 0.74)	0.0159
AA	18(18.95%)	19(17.76%)	0.62(0.28- 1.40)	0.9984
Allele				
G	118(62.11%)	115(53.74%)	Reference	
A	72(37.89%)	99(46.26%)	0.71(0.48- 1.06)	0.0893
Dominant				
GG	41(43.16%)	27(25.23%)	Reference	
AG/AA	54(56.84%)	80(74.77%)	0.45(0.25- 0.81)	0.0071
Recessive				
GG/AG	77(81.05%)	88(82.24%)	Reference	
AA	18(18.95%)	19(17.76%)	0.92(0.45- 1.89)	0.8272
rs10510097				
Genotype				

CC	49(51.58%)	70(66.04%)	Reference	
CT	41(43.16%)	35(33.02%)	1.71(0.94- 2.99)	0.4363
TT	5(5.26%)	1(0.94%)	7.14(0.81- 63.04)	0.1222
Allele				
C	139(73.16%)	175(82.55%)	Reference	
T	51(26.84%)	37(17.45%)	1.74(1.08- 2.80)	0.0230
Dominant				
CC	49(51.58%)	70(66.04%)	Reference	
CT/TT	46(48.42%)	36(33.96%)	1.83(1.03- 3.22)	0.0373
Recessive				
CC/CT	90(94.74%)	105(99.06%)	Reference	
TT	5(5.26%)	1(0.94%)	0.17(0.02- 1.50)	0.0724
rs2575735				
Genotype				
CC	68(71.58%)	64(59.81%)	Reference	
CT	25(26.32%)	36(33.64%)	0.65(0.35- 1.21)	0.6339
TT	2(2.11%)	7(6.54%)	0.27(0.05- 1.34)	0.1778
Allele				

C	161(84.74%)	164(76.64%)	Reference	
T	29(15.26%)	50(23.36%)	0.59(0.36- 0.98)	0.0404
Dominant				
CC	68(71.58%)	64(59.81%)	Reference	
CT/TT	27(28.42%)	43(40.19%)	0.59(0.33- 1.07)	0.0794
Recessive				
CC/CT	93(97.89%)	100(93.46%)	Reference	
TT	2(2.11%)	7(6.54%)	3.26(0.66- 16.07)	0.1271
rs878949				
Genotype				
CC	76(80.00%)	68(63.55%)	Reference	
CT	17(17.89%)	37(34.58%)	0.41(0.21- 0.80)	0.1548
TT	2(2.11%)	2(1.87%)	0.90(0.12- 6.53)	0.7425
Allele				
C	169(88.95%)	173(80.84%)	Reference	
T	21(11.05%)	41(19.16%)	0.52(0.30- 0.92)	0.0241
Dominant				
CC	76(80.00%)	68(63.55%)	Reference	

CT/TT	19(20.00%)	39(36.45%)	0.44(0.23- 0.83)	0.0099
Recessive				
CC/CT	93(97.89%)	105(98.13%)	Reference	
TT	2(2.11%)	2(1.87%)	0.89(0.12- 6.41)	0.9043
